# Case Report: Bacteremic respiratory tularemia causing refractory ARDS requiring VV-ECMO

**DOI:** 10.3389/fmed.2026.1965100

**Published:** 2026-10-09

**Authors:** Cynthia Wenglinski, Fabrizio Buttafuoco, Michaël Piagnerelli

**Affiliations:** 1 Department of Intensive Care Medicine, CHU Charleroi-Chimay (HUmani), Charleroi, Belgium; 2 Department of Infectious Diseases, CHU Charleroi-Chimay (HUmani), Charleroi, Belgium

**Keywords:** acute respiratory distress syndrome, bacteremia, case report, *Francisella tularensis*, tularemia, veno-venous extracorporeal membrane oxygenation

## Abstract

**Background:**

Respiratory tularemia is a rare but potentially serious manifestation of *Francisella tularensis* infection. We report a rare case of bacteremic respiratory tularemia complicated by refractory acute respiratory distress syndrome (ARDS) requiring veno-venous extracorporeal membrane oxygenation (VV-ECMO), highlighting the diagnostic challenges and therapeutic implications of this uncommon presentation.

**Case presentation:**

A 78-year-old man developed severe pneumonia following river water aspiration after a bicycle accident. After transient initial improvement, he experienced progressive respiratory deterioration despite broad-spectrum antimicrobial treatment, ultimately developing refractory ARDS requiring invasive mechanical ventilation, prone positioning and VV-ECMO. Three consecutive sets of blood cultures revealed the presence of *Francisella tularensis,* whereas bronchoalveolar lavage (BAL) cultures remained negative. No extrapulmonary manifestations of tularemia were identified. Targeted antimicrobial therapy with ciprofloxacin and gentamicin was initiated but failed to prevent a fatal outcome.

**Conclusion:**

Respiratory tularemia should be considered in patients with severe pneumonia following environmental exposure, particularly when respiratory status deteriorates despite broad-spectrum β-lactam therapy. Repeated blood cultures may establish the diagnosis despite negative BAL cultures. Early recognition and prompt initiation of effective antimicrobial therapy are essential, as β-lactam antibiotics are clinically ineffective against *F. tularensis*. This case also illustrates the potential severity of respiratory tularemia, which may progress to refractory ARDS requiring VV-ECMO.

## Introduction

*Francisella tularensis* is a facultatively intracellular Gram-negative coccobacillus responsible for a rare zoonosis. Although ulceroglandular forms are the most common, respiratory involvement is less frequent and is associated with some of the most severe clinical manifestations. Its nonspecific clinical presentation may delay diagnosis and the initiation of effective antimicrobial treatment. We report a case of bacteremic respiratory tularemia complicated by refractory acute respiratory distress syndrome (ARDS) requiring veno-venous extracorporeal membrane oxygenation (VV-ECMO), a presentation rarely reported in the literature.

## Case report

We report the case of a 78-year-old man, who was functionally independent and whose medical history was limited to gastro-oesophageal reflux treated with pantoprazole, a radical prostatectomy and inguinal hernia repair performed approximately 20 years previously. He was admitted following a cycling accident during which he was found submerged in a river, with documented aspiration of river water. On admission, he was conscious but hypothermic, with a body temperature of 34 °C.

The patient was initially admitted to the intensive care unit (ICU) for acute hypoxemic respiratory failure requiring non-invasive ventilation (NIV) for approximately 24 h. Chest imaging revealed diffuse bilateral pulmonary infiltrates (Figure 1), associated with a moderate inflammatory response with a C-reactive protein (CRP) level of 56 mg/L. Empirical antimicrobial treatment with amoxicillin-clavulanate (1 g every 6 h) was initiated for suspected aspiration pneumonia. A multiplex PCR test performed on a sputum sample detected *Escherichia coli*, *Klebsiella aerogenes*, *Klebsiella oxytoca* and *Proteus mirabilis*. His respiratory condition improved rapidly, allowing NIV to be discontinued and the patient to be transferred to the cardiology department on day 2, whilst receiving oxygen at 2 L/min via a nasal cannula.

**Figure 1:**
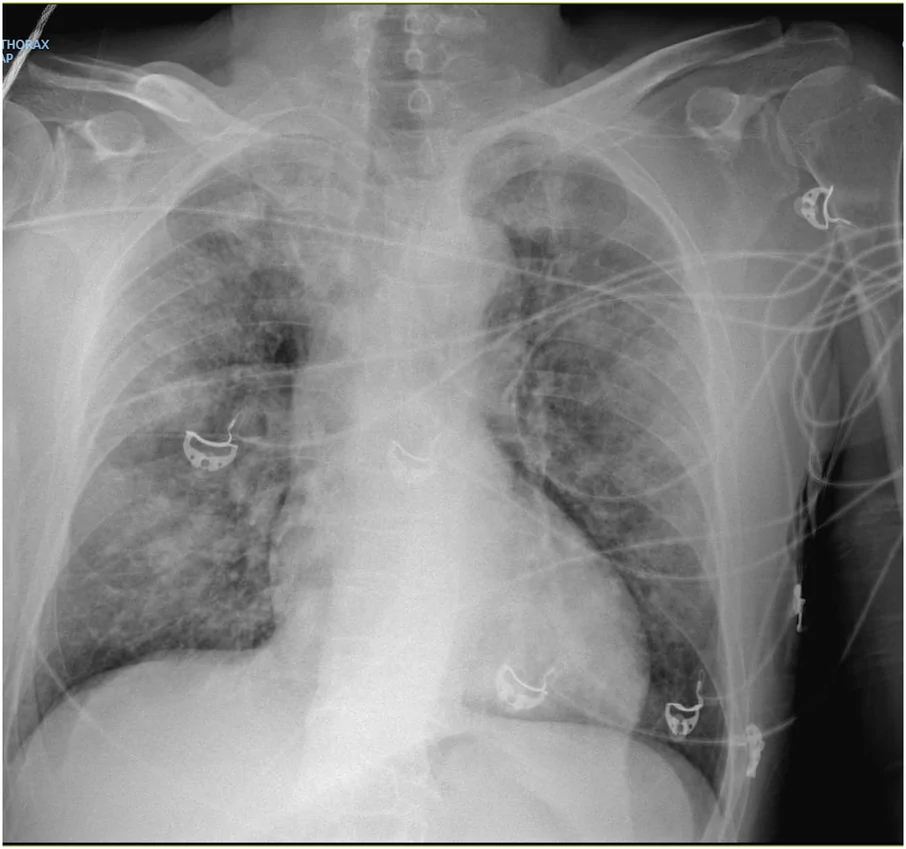
Chest computed tomography performed on admission showing bilateral diffuse ground-glass opacities and multifocal consolidations predominantly involving the lower lobes, consistent with severe pneumonia.

Because of this rapid clinical and respiratory improvement during the first 48 h, no BAL was performed during the initial ICU stay.

From day 3 onwards, the patient's respiratory condition gradually deteriorated. By day 4, oxygen requirements had increased to 5 L/min via a nasal cannula. Arterial blood gas analysis revealed a PaO_2_ of 66 mmHg, corresponding to a PaO_2_/FiO_2_ ratio of approximately 165. At this stage, the respiratory rate remained below 20 breaths per minute, with no hyperlactatemia, but the inflammatory response had markedly worsened, with CRP rising from 56 to 305 mg/L.

In view of the worsening respiratory and inflammatory status, antimicrobial therapy was escalated to piperacillin-tazobactam on day 5. During this period, repeated conventional blood cultures remained negative. No further sputum samples could be obtained, as the patient no longer had productive cough. No BAL was performed during this second phase of deterioration. Given the initial results of the multiplex PCR identifying several Gram-negative microorganisms and the initially suspected aspiration-related bacterial pneumonia, no specific molecular testing for *F. tularensis* was carried out at this stage.

Despite broad-spectrum antimicrobial treatment, respiratory failure continued to worsen. Meropenem was initiated on day 7. The patient subsequently required a non-rebreathing mask with an FiO_2_ of 60% and developed overt respiratory distress, with a respiratory rate of up to 30 breaths per minute and sinus tachycardia of approximately 95 beats per minute. Neither high-flow nasal oxygen therapy nor NIV was used in the cardiology department.

On day 8, he was readmitted to the intensive care unit for severe hypoxemic respiratory failure. A brief trial of NIV was conducted with an FiO_2_ of 55%, a positive end-expiratory pressure (PEEP) of 8 cmH_2_O and pressure support of 5 cmH_2_O. Arterial blood gas analysis revealed a pH of 7.51, a PaCO_2_ of 28 mmHg and a PaO_2_ of 60 mmHg, corresponding to a PaO_2_/FiO_2_ ratio of 109. Clarithromycin (500 mg every 12 h) was added to meropenem.

In view of persistent severe hypoxemia, the patient was intubated, deeply sedated and treated with continuous neuromuscular blockade using cisatracurium. Lung-protective mechanical ventilation was initiated. At an FiO_2_ of 70%, the ventilatory settings comprised a respiratory rate of 15 breaths per minute, a tidal volume of approximately 200 mL, PEEP of 5 cmH_2_O, a plateau pressure of 20 cmH_2_O and a driving pressure of 15 cmH_2_O. Respiratory system compliance was markedly reduced, at approximately 13 mL/cmH_2_O. Increasing PEEP did not result in any significant improvement in oxygenation.

The patient was placed in the prone position. After approximately 2 h of prone positioning, severe refractory hypoxemia persisted, with a PaO_2_/FiO_2_ ratio of approximately 80 despite an FiO_2_ of 100%. Given refractory ARDS despite lung-protective ventilation, neuromuscular blockade, PEEP optimization and prone positioning, VV-ECMO was initiated (Figure 2).

**Figure 2:**
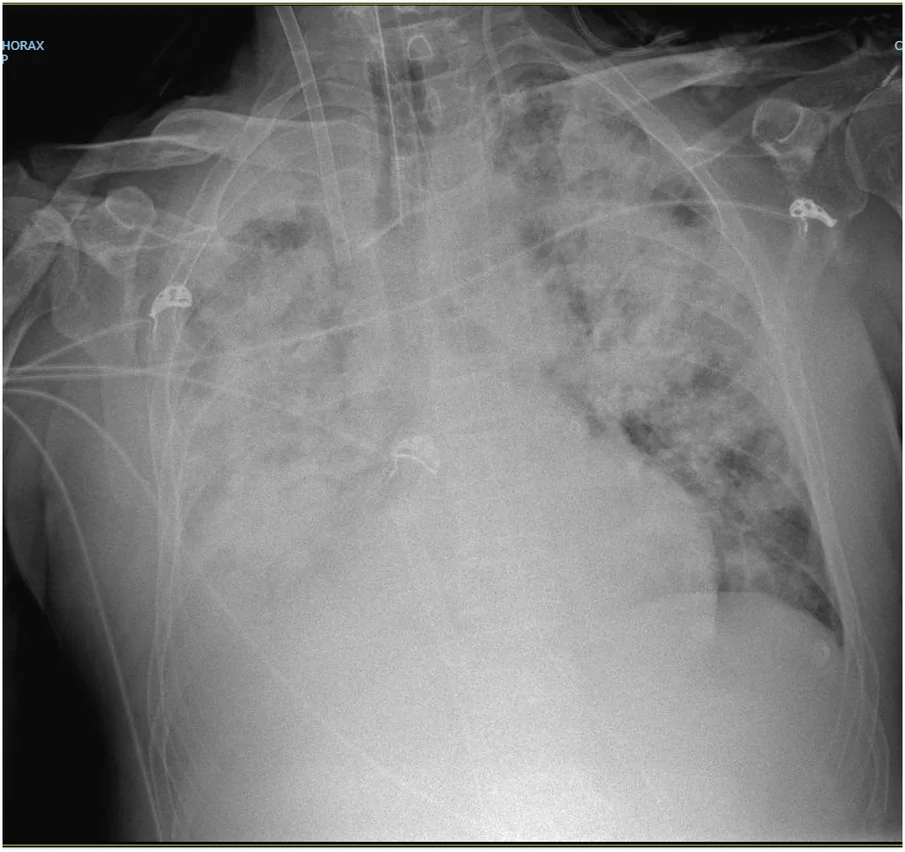
Chest radiograph obtained after initiation of veno-venous extracorporeal membrane oxygenation demonstrating persistent bilateral diffuse pulmonary infiltrates despite lung-protective mechanical ventilation.

A BAL and three sets of blood cultures were obtained following readmission to the intensive care unit. Given the severity and rapid progression of respiratory failure, a comprehensive infectious disease work-up, including screening for opportunistic infections associated with immunocompromised states, was carried out and yielded negative results. No systemic corticosteroids were administered during the course of the illness.

Three consecutive blood-culture sets subsequently grew *Francisella tularensis*, whilst the BAL cultures remained negative. All blood cultures performed prior to readmission to the intensive care unit had remained sterile. Physical examination revealed no skin or oral ulcers, lymphadenopathy or other classical extrapulmonary manifestations of tularemia.

Following the microbiological identification of *F. tularensis*, meropenem was discontinued on day 10 and targeted antimicrobial therapy combining ciprofloxacin (400 mg three times daily) and gentamicin (6 mg/kg/day) was initiated.

Despite targeted antimicrobial treatment and maximal supportive care, severe hypoxemia persisted on VV-ECMO. The patient developed disseminated intravascular coagulation associated with severe thrombocytopenia, with a platelet nadir of 40 × 10⁹/L, normocytic anemia requiring three red blood cell concentrate transfusions, as well as diffuse hemorrhagic manifestations affecting the VV-ECMO cannulation sites and the oral cavity. Anti-PF4 antibodies were negative and there were no laboratory signs of significant ECMO-related hemolysis, with total bilirubin, lactate dehydrogenase and haptoglobin levels within normal limits. Therapeutic anticoagulation was discontinued due to persistent bleeding.

The patient died on day 11 due to persistent refractory respiratory failure. No septic shock or acute kidney injury was observed, and no extrapulmonary manifestations of tularemia were identified. The full clinical course is summarized in Figure 3.

**Figure 3:**
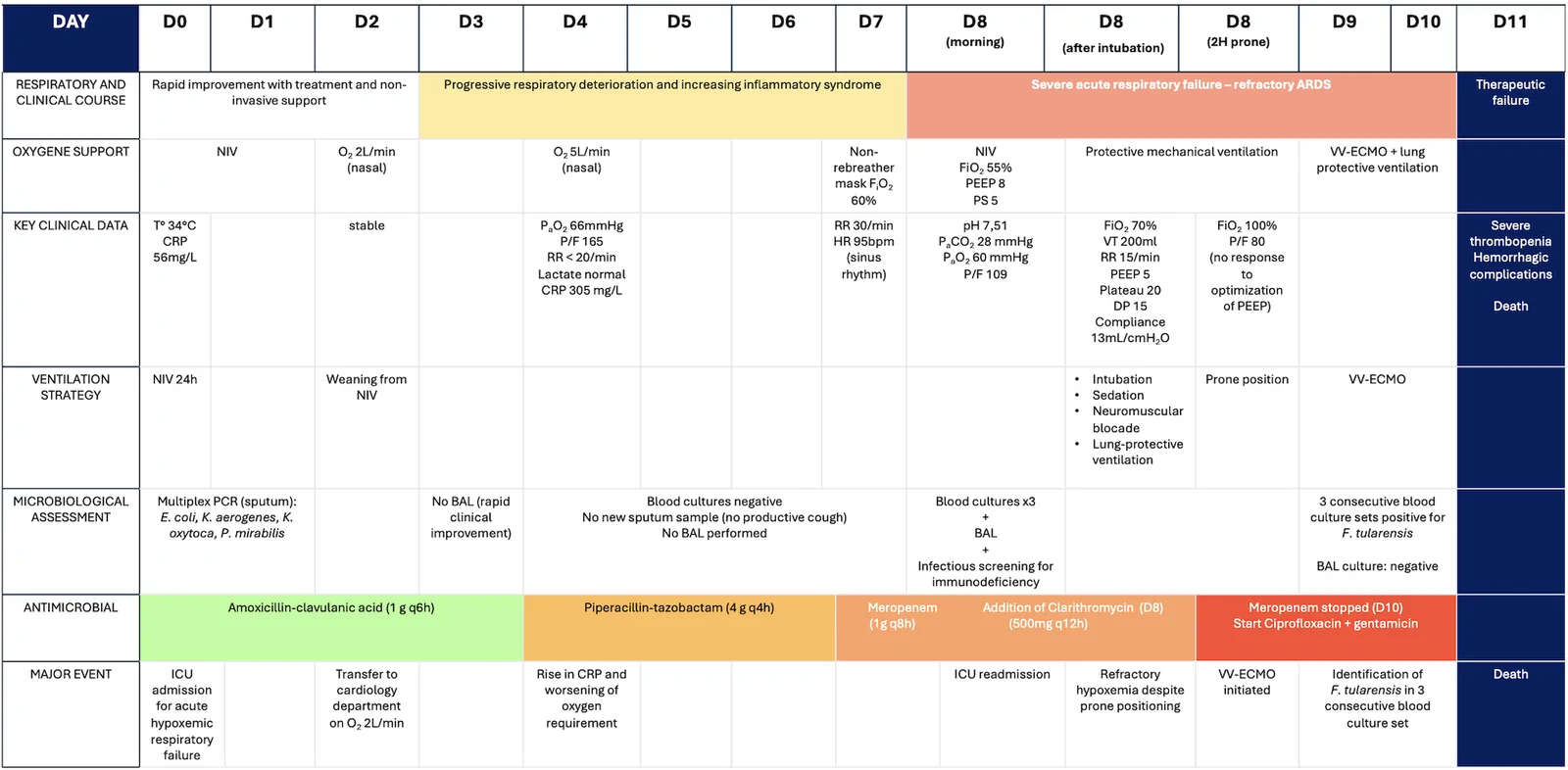
Clinical timeline of bacteremic respiratory tularemia progressing to refractory ARDS requiring VV-ECMO. The timeline summarizes the evolution of respiratory failure, oxygen requirements, major clinical and microbiological findings, antimicrobial therapy, ventilatory strategies, and initiation of VV-ECMO. Legend: BAL, bronchoalveolar lavage; CRP, C-reactive protein; NIV, non-invasive ventilation; P/F, PaO_2_/FiO_2_ ratio; PEEP, positive end-expiratory pressure; VV-ECMO, veno-venous extracorporeal membrane oxygenation.

## Discussion

*Francisella tularensis* is an intracellular, Gram-negative coccobacillus responsible for a rare zoonosis, the main reservoirs of which are small mammals. Transmission to humans occurs via several routes, including arthropod bites, direct contact with infected animals, ingestion of contaminated food or water, and inhalation of contaminated aerosols (1).

The ulceroglandular and glandular forms account for the majority of cases, whilst the pneumonic and typhoidal forms are significantly less common but are associated with a higher risk of severe complications and death (1, 3).

Diagnosing respiratory tularemia is particularly challenging due to its nonspecific clinical and radiological presentation. The absence of skin lesions or lymphadenopathy does not rule out the diagnosis. In patients presenting with severe pneumonia or ARDS without a clearly established microbiological etiology, a thorough assessment of potential environmental exposures is therefore essential (3, 4). Chest computed tomography findings are nonspecific and may include subpleural pulmonary opacities, mediastinal or hilar lymphadenopathy, and pleural effusion (5).

Microbiological diagnosis relies on culture, specific molecular tests and serology. Culture remains an important diagnostic method, although its sensitivity is limited and the slow and fastidious growth of *F. tularensis* can delay its identification. Specific PCR testing may enable earlier microbiological confirmation, but *F. tularensis* is not routinely included in standard multiplex respiratory PCR panels. Serology also has limitations during the early phase of infection, as antibodies generally become detectable only two to three weeks after the onset of symptoms (1, 3, 6).

Bacteremia caused by *F. tularensis* is rare, owing both to the intracellular nature of the organism and to the difficulties involved in culturing it. Schepens et al. reported a case of *F. tularensis* subsp. *holarctica* bacteremia in an immunocompetent patient (7), whilst a Swedish cohort study showed that bacteremic tularemia was associated with older age, a greater burden of comorbidities and a longer hospital stay (5). In the present case, the isolation of *F. tularensis* from three consecutive blood-cultures sets was particularly noteworthy, especially as the concurrent BAL cultures remained negative.

β-lactam antibiotics, including carbapenems, are clinically ineffective against *F. tularensis* despite occasional *in vitro* activity (1). Current guidelines recommend aminoglycosides, particularly gentamicin, for severe tularemia, whilst fluoroquinolones represent another effective therapeutic option and may be used in combination in severe cases (1). The available data, although largely observational, consistently support the use of aminoglycosides, fluoroquinolones and tetracyclines as active agents (2). Fatal cases nevertheless occur despite appropriate antimicrobial treatment, particularly when active treatment is initiated late, suggesting that the time to effective antimicrobial therapy may be an important prognostic factor (1, 2).

Respiratory tularemia can progress to severe pneumonia, sepsis, ARDS and multiorgan failure despite appropriate antimicrobial treatment. Recent reports have described cases of pneumonic tularemia progressing to septic shock or severe ARDS (8, 9).

In our patient, respiratory failure progressed to severe refractory hypoxemia. During the period of respiratory deterioration that occurred in the cardiology unit, the patient experienced marked respiratory distress, with a respiratory rate reaching up to 30 breaths per minute, while receiving oxygen via a non-rebreathing mask, without transitioning to high-flow nasal oxygen therapy or NIV. The lack of advanced noninvasive respiratory support during this period may therefore have allowed sustained excessive inspiratory effort, which could theoretically have contributed to further lung injury through mechanisms of patient self-inflicted lung injury (P-SILI). Following ICU readmission, severe hypoxemia persisted despite lung-protective mechanical ventilation, neuromuscular blockade, PEEP optimization and prone positioning, ultimately requiring VV-ECMO. To our knowledge, the use of VV ECMO for refractory ARDS associated with bacteremic respiratory tularemia has not previously been reported. This case also illustrates the diagnostic challenges associated with severe respiratory tularemia when initial microbiological findings suggest an alternative etiology. In retrospect, the combination of documented river-water aspiration and secondary respiratory deterioration despite treatment with broad-spectrum β-lactams might have prompted earlier consideration of uncommon environmental pathogens, including *F. tularensis,* and earlier identification would have allowed earlier initiation of effective antimicrobial therapy. However, whether this would have altered the outcome in the setting of rapidly progressive refractory ARDS remains uncertain.

Several limitations should be acknowledged. No serological confirmation of tularemia was obtained and, although documented river-water aspiration represents a plausible environmental exposure, the source of the *F. tularensis* infection cannot be definitively established. Furthermore, specific molecular testing for *F. tularensis* was not performed during the early course of the disease. Nevertheless, the isolation of *F. tularensis* from three consecutive blood-cultures sets provides strong microbiological evidence supporting the diagnosis.

## Conclusion

Respiratory tularemia should be considered in the differential diagnosis of severe pneumonia or ARDS following relevant environmental exposure, particularly when respiratory status worsens despite treatment with broad-spectrum β-lactam antibiotics. Diagnosis may prove difficult due to nonspecific clinical and radiological findings and the limitations of routine respiratory microbiological testing. Repeated blood cultures may provide decisive diagnostic evidence, even when BAL cultures are negative.

This case demonstrates that bacteremic respiratory tularemia can progress to refractory ARDS requiring VV-ECMO. Earlier identification of *F. tularensis* might have enabled more timely initiation of effective antimicrobial therapy, although whether this would have altered the fatal outcome in this rapidly progressing case remains uncertain.

## Data Availability

The original contributions presented in the study are included in the article/Supplementary Material, further inquiries can be directed to the corresponding author.
